# Dynamic RNA Fitness Landscapes of a Group I Ribozyme during Changes to the Experimental Environment

**DOI:** 10.1093/molbev/msab373

**Published:** 2022-01-10

**Authors:** Gianluca Peri, Clémentine Gibard, Nicholas H Shults, Kent Crossin, Eric J Hayden

**Affiliations:** 1 Biomolecular Sciences Graduate Programs, Boise State University, Boise, ID, USA; 2 Department of Biological Science, Boise State University, Boise, ID, USA

**Keywords:** fitness landscape, gene–environment interactions, molecular evolution, ribozyme, noncoding RNA

## Abstract

Fitness landscapes of protein and RNA molecules can be studied experimentally using high-throughput techniques to measure the functional effects of numerous combinations of mutations. The rugged topography of these molecular fitness landscapes is important for understanding and predicting natural and experimental evolution. Mutational effects are also dependent upon environmental conditions, but the effects of environmental changes on fitness landscapes remains poorly understood. Here, we investigate the changes to the fitness landscape of a catalytic RNA molecule while changing a single environmental variable that is critical for RNA structure and function. Using high-throughput sequencing of in vitro selections, we mapped a fitness landscape of the *Azoarcus* group I ribozyme under eight different concentrations of magnesium ions (1–48 mM MgCl_2_). The data revealed the magnesium dependence of 16,384 mutational neighbors, and from this, we investigated the magnesium induced changes to the topography of the fitness landscape. The results showed that increasing magnesium concentration improved the relative fitness of sequences at higher mutational distances while also reducing the ruggedness of the mutational trajectories on the landscape. As a result, as magnesium concentration was increased, simulated populations evolved toward higher fitness faster. Curve-fitting of the magnesium dependence of individual ribozymes demonstrated that deep sequencing of in vitro reactions can be used to evaluate the structural stability of thousands of sequences in parallel. Overall, the results highlight how environmental changes that stabilize structures can also alter the ruggedness of fitness landscapes and alter evolutionary processes.

## Introduction

Fitness landscapes are maps of how changes to biological sequences (genotypes) result in phenotypic or fitness differences at the genome or individual gene level. The fitness landscapes of entire genomes are too vast to explore comprehensively, and experimental approaches have focused on the investigation of smaller local fitness landscapes (de Visser and Krug 2014). The genotypes in these local fitness landscapes are chosen from combinations of mutations that have been identified as important based on the outcomes of natural or experimental evolution, or from bioinformatics approaches. Molecular fitness landscapes result from the study of numerous combinations of mutations within a single gene product, such as a protein or a noncoding RNA molecule. For example, the fitness landscape of beta lactamase proteins has been used to study the evolution of antibiotic resistance (Weinreich et al. 2006), and RNA fitness landscapes have been used to study the emergence of new RNA structures (Jiménez et al. 2013; Bendixsen et al. 2019; Pressman et al. 2019). The study of molecular fitness landscapes has helped explain the evolutionary constraints to optimizing gene functions, which have important implications for understanding and predicting evolutionary outcomes (Wagner 2011; Ferretti et al. 2018). The rich data sets are providing opportunities to interpret past evolution (Yang et al. 2019; Bendixsen et al. 2021), predict future evolution (de Visser and Krug 2014), and more effectively harness molecular evolution in the laboratory (Ogden et al. 2019; Townshend et al. 2021).

Epistasis is an important feature of molecular fitness landscapes. Epistasis in this context refers to a nonadditive effect when two or more mutations are combined, such that the fitness effect of a mutation depends on the presence or absence of other mutations. In extreme cases (sign epistasis) the effect of a mutation can be beneficial or deleterious depending on the presence or absence of other mutations. In terms of the topography of fitness landscapes, sign epistasis causes ruggedness—multiple peaks and valleys in the landscape—where peaks are commonly defined as genotypes where all the analyzed mutational neighbors have lower fitness (Poelwijk et al. 2011). Landscape ruggedness is thought to constrain evolution by making some sequential orders of mutations unlikely to occur, even if the final mutational combination is beneficial (Ferretti et al. 2018). To evolve away from a peak genotype toward higher fitness may require the preservation of a deleterious mutation in the population long enough for a second mutation to improve function, or the simultaneous occurrence of two or more specific mutations. If a genotype is a peak in the local fitness landscape, random mutations are unlikely to improve the function, and therefore evolution is less likely to preserve the multiple mutational changes required to generate a high-fitness genotype. Numerous experimental data sets have demonstrated that molecular fitness landscapes are in fact rugged with multiple suboptimal peaks caused by the epistatic interactions of mutations (Szendro et al. 2013).

Another important aspect of fitness landscapes is the interaction between genetic differences and the environment. Protein and RNA functions are sensitive to numerous components of the cellular environment, such as ionic strength, pH, temperature, the expression level of other genes, and metabolite concentrations. Experimental investigations of RNA and protein landscapes in yeast have shown that altering the growth conditions can change epistatic interactions, and the topography of the molecular fitness landscape (Li and Zhang 2018; Flynn et al. 2020). The constant fluctuation in natural environments means that fitness landscapes are in fact dynamic. Measuring changes to the effects of numerous mutations in different environments remains a challenge for characterizing fitness landscapes and for using the information for various applications.

Here, we study fitness landscapes of a noncoding RNA in a cell-free biochemical reaction while experimentally changing the magnesium ion concentration. RNA folding and function are strongly dependent upon the concentration of ions in the environment. Magnesium is particularly effective at stabilizing the native folds of RNA molecules, and the amount of magnesium required is related to the thermodynamic stability of the native state (Draper 2008). Group I self-splicing introns have been a foundational model of RNA structure and folding in part because the catalytic activity of these ribozymes is a very sensitive readout for native tertiary structure (Rangan et al. 2004; Sinan et al. 2011). For our experiments, we chose to study mutational variants of the group I self-splicing intron from the bacterium *Azoarcus* (Tanner and Cech 1996; GenBank accession number DQ103524.1). This ribozyme is well characterized in terms of reaction kinetics (Gleitsman and Herschlag 2014), folding (Rangan et al. 2004), and structure (Stahley and Strobel 2005), providing guidance for reaction conditions and for interpreting results. In addition, the *Azoarcus* ribozyme has been engineered to study the origin of life (Vaidya et al. 2012), and to deliver mRNA repairing gene therapies (Dolan and Müller 2014). Importantly, the reverse-splicing reaction of this ribozyme allows for the enrichment of molecular variants based on how much reacted during a biochemical assay (Beaudry and Joyce 1992). Numerous ribozyme sequence variants can be cosynthesized and studied in parallel. The enrichment of each sequence from in vitro selection can be detected by high-throughput sequencing (Hayden et al. 2011, 2015). This experimental system allows the study of numerous genetic differences while systematically altering a single isolated environmental variable.

**Fig. 1.**
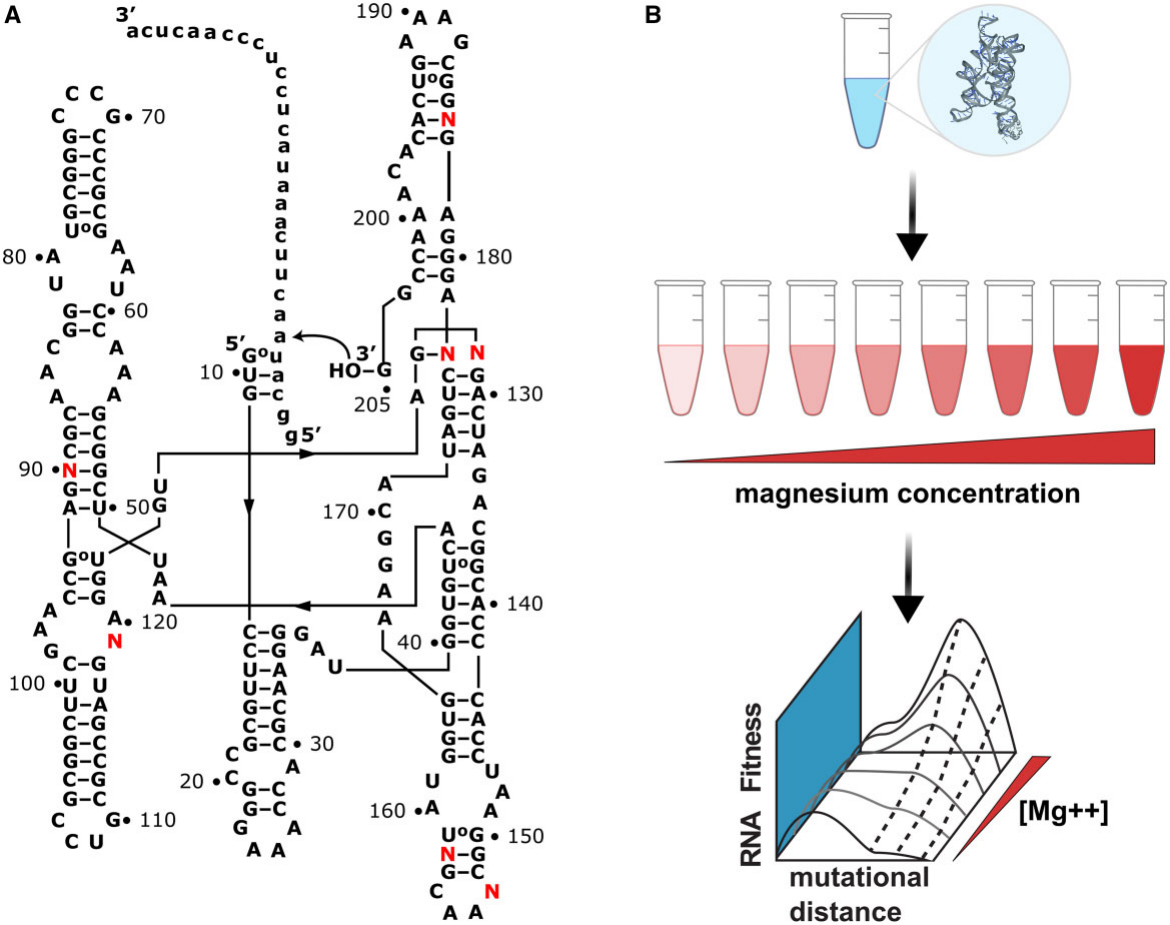
In vitro selection of ribozyme activity under different magnesium conditions. (*A*) The secondary structure of the *Azoarcus* ribozyme with nucleotides numbered according to intronic nucleotides. Randomized nucleotides (*N*) are highlighted in red. The RNA substrate used to select for reverse-splicing activity is shown as lowercase. The curved arrow indicates the reaction used for selection, which results in a portion of the substrate appended to the 3′-end of active ribozymes. (*B*) Experimental concept. The in vitro transcribed ribozyme library (blue tube, crystal structure 1ZZN) is reacted in different magnesium concentrations (red tubes). High-throughput sequence data are used to generate RNA fitness landscapes by determining the *RNA Fitness* (enrichment/depletion) of each ribozyme variant at each magnesium concentration [Mg^++^].

The combinations of mutations studied here were chosen based on mutational effects observed in a previous study where random mutations were introduced into the *Azoarcus* ribozyme followed by in vitro selection for reverse-splicing activity under low (2 mM) or high (25 mM) MgCl_2_ concentrations (Hayden et al. 2015). The selection enriched for ribozymes that were active under each condition. Following this selection, several sequences were isolated and studied for their sensitivity to MgCl_2_ by measuring their activity relative to the wild-type ribozyme at both 2 mM and 25 mM MgCl_2_. This analysis can be found in the Supporting Information of the previous publication (Hayden et al. 2015). To identify the nucleotide positions studied herein, we chose from this previous analysis one sequence with high Mg^2+^ sensitivity, one sequence with low Mg^2+^ sensitivity and one sequence with intermediate Mg^2+^ sensitivity from the previous analysis. The high sensitivity sequence (E27-1) showed very low activity at 2 mM MgCl_2_ and high activity at 25 mM MgCl_2_ and contained the mutations C90G, A128C, and C177U. The sequence with low MgCl_2_ sensitivity (D45-5) showed high activity at both 2 mM and 25 mM MgCl_2_ and contained the mutations A153G and U184C. The third sequence (E27-12) showed intermediate activity at 2 mM Mg and high activity at 25 mM Mg and contained the mutations A119G and C158U. To make the library used here, we randomized all seven nucleotide positions (90, 119, 128, 153, 158, 177, 184) where mutations were found in these three sequences (fig. 1*A*). This resulted in a library of 4^7^=16,384 different sequences (four possible nucleotides at seven positions) and included numerous mutations of unknown function. We expected that the mutations would show a range of magnesium dependence and provide opportunities for magnesium induced changes to the topography of the fitness landscape. Seven random nucleotide positions were chosen to achieve a number of sequence variants that could be realistically studied under multiple magnesium concentrations, in triplicate, and still have full sequencing coverage at adequate depth using current high-throughput sequencing platforms.

## Results

To determine the fitness of all 16,384 RNA sequences in eight different magnesium concentrations, the RNA pool was allowed to react with a reverse-splicing substrate under a given magnesium concentration, and the reacted ribozymes were amplified by reverse-transcription PCR (RT-PCR). The frequency of each sequence in the pools after selection was compared with their frequency before selection as determined by high-throughput sequencing (see Materials and Methods). High-throughput sequencing yielded sufficient read coverage for each genotype in the pools (supplementary fig. 2, Supplementary Material online). We define ribozyme fitness based on the change in population frequency of each sequence because the relative activity of each ribozyme sequence largely determines how much of that sequence reacts and then gets amplified by RT-PCR (supplementary fig. 1, Supplementary Material online). Sequences with a larger increase in frequency have higher fitness, and those that decrease in frequency have low fitness. The frequency of each sequence was normalized to the frequency of the wild-type *Azoarcus* ribozyme at that magnesium concentration. The activity of the wild-type ribozyme at each magnesium concentration was also determined by PAGE (supplementary fig. 4, Supplementary Material online). Fitness values were highly correlated between replicates (supplementary fig. 3, Supplementary Material online) and with low coefficients of variation that increased for the sequences with the lowest activity (fitness<0.15) (supplementary fig. 11, Supplementary Material online).

**Fig. 2.**
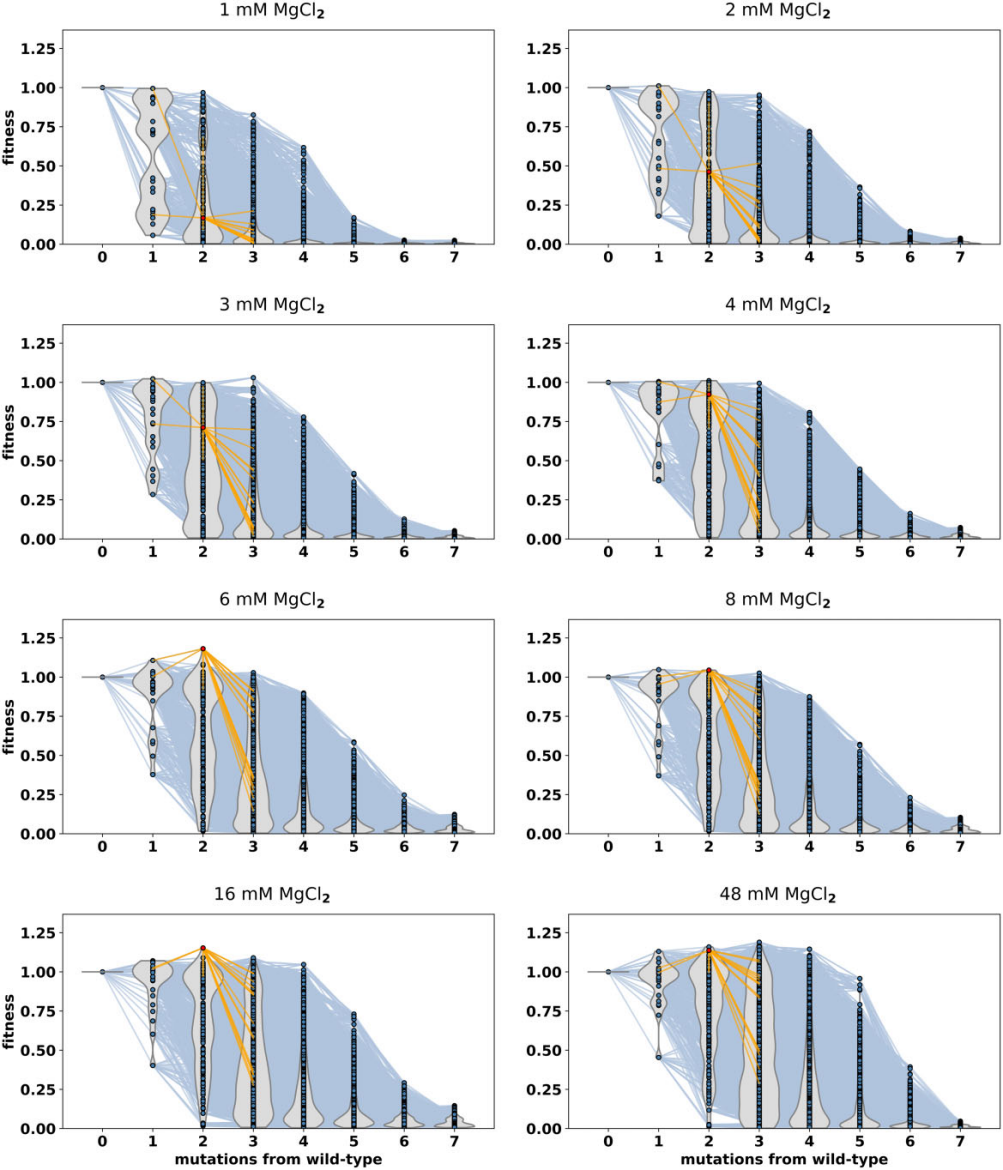
The RNA fitness landscapes under different magnesium ion concentrations. For each graph, nodes represent individual sequences plotted with mutational distance (*x* axis) and fitness (*y* axis) relative to the wild-type ribozyme. Each node shows the average fitness of three experimental replicates. Nodes are connected by a blue edge if the two sequences differ by a single-nucleotide difference. Violin plots (gray) represent the distribution of fitness values at each mutational distance. The magnesium chloride concentration (MgCl_2_) used for each data set is shown above each graph. A sequence is highlighted (yellow edges) to illustrate magnesium induced changes to fitness.

**Fig. 3.**
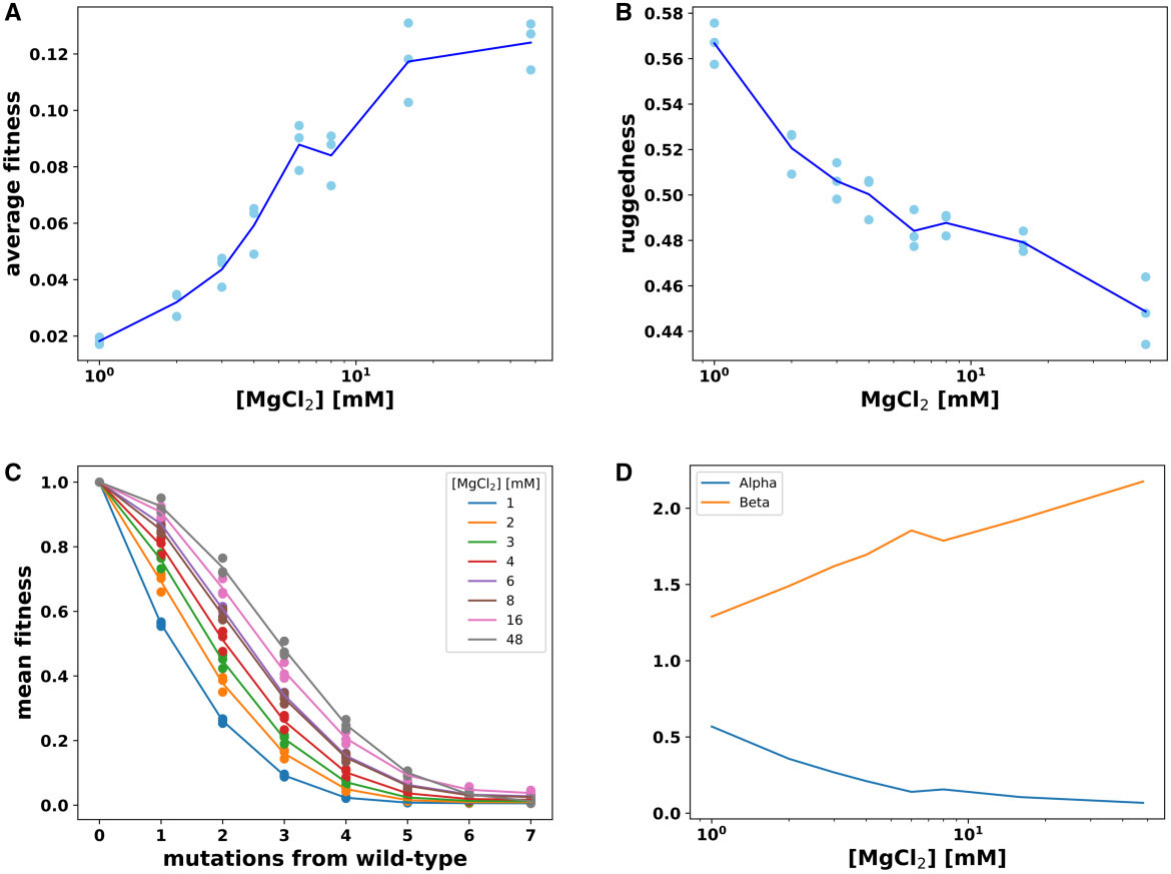
Magnesium induced changes to the fitness landscapes. (*A*) The mean fitness of all variants in the library, relative to wild-type, at each magnesium ion concentration ([MgCl_2_]). Three data points at each concentration represent experimental replicates. The blue line traces the mean value of the triplicates at each magnesium ion concentration. (*B*) Landscape ruggedness calculated as ruggedness=2(*f*_RS_)+*f*_S_, where *f*_RS_ is the fraction of reciprocal sign epistasis and *f*_S_ is the fraction of sign epistasis for all pairs of mutations. (*C*) Mean fitness plotted as a function of mutational distance from wild-type. Data points represent the mean of the fitness values of all genotypes with that many mutations at that magnesium concentration. Lines are best-fit curves to the equation *w*(*n*)=exp(−α*n*^β^). (*D*) Effect of magnesium concentration on the exponential decay parameter α and directional epistasis parameter β from the curve fitting.

To visually evaluate changes in the ribozyme fitness landscape caused by different magnesium concentrations, fitness was plotted as a function of mutational distance from the wild-type sequence (fig. 2). We also created an interactive data analysis tool that was used to explore the data (available at https://haydenlab.gitlab.io/azo). The results showed that relative fitness of many sequences in the library increased with increasing magnesium concentration (supplementary figs. 6, 7, and 10, Supplementary Material online). Both the maximum fitness observed and the average fitness observed increased with increasing magnesium concentration (fig. 3). The number of sequences with fitness higher than wild-type (fitness>1) also increased with higher magnesium. However, several sequences with higher than wild-type fitness at higher concentrations of magnesium had much lower fitness at low magnesium (one of these genotypes is highlighted in fig. 2). Specifically, several sequences with three or four mutations were more active than wild-type only at the two highest magnesium concentrations (16 and 48 mM magnesium). These results suggest that certain combinations of mutations can be both beneficial for ribozyme activity yet destabilizing for the ribozyme structure, requiring more magnesium to appear as beneficial.

Next, we asked whether the ruggedness of the landscapes changed with magnesium concentration. As a first approach to measure ruggedness, we determined the frequency of sign and reciprocal sign epistasis in each data set (Szendro et al. 2013). Sign epistasis occurs when one individual mutation is deleterious but has a beneficial effect in the presence of a second already beneficial mutation. Reciprocal sign epistasis occurs when both individual mutations are deleterious, but combined they are beneficial. This severe form of epistasis is the cause of peaks and valleys in fitness landscapes. Each landscape was divided into four-genotype subgraphs (squares) containing a starting genotype, two single mutants of this genotype and the genotype with both individual mutations combined. The fitness of the four genotypes in the square was used to identify instances of epistasis. Specifically, the fraction of squares with sign epistasis and reciprocal sign epistasis was determined. We used the formula ruggedness=2(*f*_RS_)+*f*_S_, where *f*_RS_ is the fraction of reciprocal sign epistasis and *f*_S_ is the fraction of sign epistasis (Wu et al. 2016). The data showed a significant negative correlation between ruggedness and magnesium concentration (Spearman correlation coefficient *r*=−0.94, *P*=2.15×10^−11^) (fig. 3*B*). With increasing magnesium there was a decrease in the fraction of squares that exhibit sign epistasis and reciprocal sign epistasis. We also assessed changes to ruggedness using the roughness to slope ratio, *r*/*s* (Carneiro and Hartl 2010; Szendro et al. 2013). This approach measures the level of epistasis as the deviation from a purely additive model by fitting a multidimensional linear model to the data. We found that *r*/*s* also decreased with increasing magnesium (supplementary fig. 9, Supplementary Material online). Both measurements indicate that increasing magnesium decreased ruggedness in this local ribozyme fitness landscape.

To further characterize the fitness landscapes, we analyzed how the interactions between mostly deleterious mutational effects changed with magnesium concentration on average. For this analysis, we fit the equation *W*(*n*)=exp(−α*n*^β^) to the fitness (*W*) by mutational distance (*n*) data at each magnesium concentration. This equation has been previously used to detect the average deleterious effect of mutations (α) and any predominant direction of epistasis (β) (Bendixsen et al. 2017). We found that these parameters show an inverse relationship in response to magnesium concentration (fig. 3*D*). The deleterious effect of individual mutations α decreased with increasing magnesium, which is expected as magnesium shields the destabilizing effect of most mutations. Values for β were positive at all magnesium concentrations, indicating a predominance of negative epistasis where mutations tend to have a more deleterious effect in combination than what would be expected if the individual effects were additive. Values for β increased with increasing magnesium, indicating that while higher magnesium reduces individual mutational effects, the higher magnesium concentrations studied could not completely shield the destabilizing effect of increasing numbers of mutations. The inverse relationship between the average effect of individual mutations and their combined effects has been observed previously in antibiotic resistance proteins where antibiotic concentration was varied (Bershtein et al. 2006). Our results, observed under varying magnesium concentrations, further support the generality of this phenomenon.

**Fig. 4.**
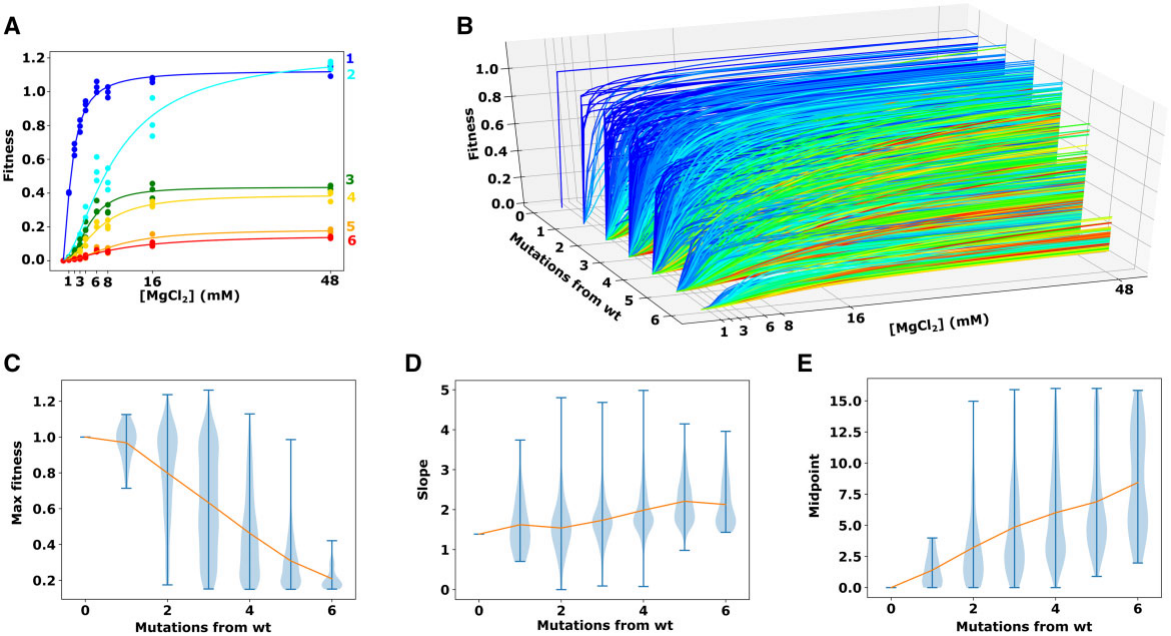
Magnesium induced changes to individual ribozyme variants. (*A*) Example ribozymes with different numbers of mutations. Curves are the Hill equation fit to the fitness data. The color of the lines represents the genetic distance from wild-type, which is indicated next to each curve. (*B*) Curve fits to all sequences in the data, plotted by mutational distance. The line colors represent the midpoint of each curve (gradient from blue to red as the midpoint moves away from 1 mM). Only the curves with max fitness between 0.15 and 3, slope lower than 5, and midpoint lower than 16 were included. Distributions of maximum fitness (*C*), hill slopes (*D*), and midpoints from the Hill equation curve fitting, with sequences categorized by mutational distance from wild-type.

Magnesium titrations have been used extensively to understand RNA folding because the magnesium required to achieve the native folded state is related to the thermodynamics of the unfolded to folded transition. In our data, individual active ribozymes showed a transition from lower to higher fitness with magnesium, and we fit the Hill equation to these data (fig. 4). We were able to fit a curve and extract best fit parameters for all active ribozymes that achieved a maximum fitness greater than fitness=0.15. We found that on average, the midpoint of the transition tends to increase as a function of mutational distance from wild-type. This further indicates that most mutations have a destabilizing effect, and on average sequences with more mutations require more magnesium to stabilize the native, active fold. The results indicate that the effect of magnesium titrations on RNA folding can be evaluated by deep sequencing of in vitro selections, which can enable the study of RNA structure and folding of many different RNA sequences in a highly parallel fashion.

The observed changes to the ruggedness of the fitness landscapes suggest that changes in magnesium could alter evolutionary processes. For example, the general smoothing of the landscapes at higher magnesium indicates that more mutational pathways to higher fitness become “accessible,” which could promote more rapid evolutionary adaptation. However, evolving populations are subject to random mutations and therefore encounter both optimal and suboptimal intermediates in a stochastic fashion. To better understand the evolutionary consequences of the changing RNA landscapes, we used computational simulations of populations of ribozymes competing for survival. Each simulated evolution is run for a set number of “generations.” For each generation, a population of 1000 individuals is chosen from the genotypes present in the previous generation. Individuals are chosen at random to reproduce, but the probability of reproduction is relative to the fitness of that genotype in the given magnesium environment (see Materials and Methods). High-fitness genotypes are more likely to reproduce. Each individual that reproduces also has a random chance of acquiring a single mutation that converts it to a neighboring genotype. If this genotype is of higher fitness, the higher fitness genotype has a higher probability of survival in the subsequent generations. Through this process of reproduction with mutation, the population tends to increase in fitness as higher fitness genotypes emerge and replace lower fitness genotypes. These simulations have the advantage of taking into account both the frequency and the magnitude of epistatic interactions while also allowing stochastic processes that mimic natural evolution.

**Fig. 5.**
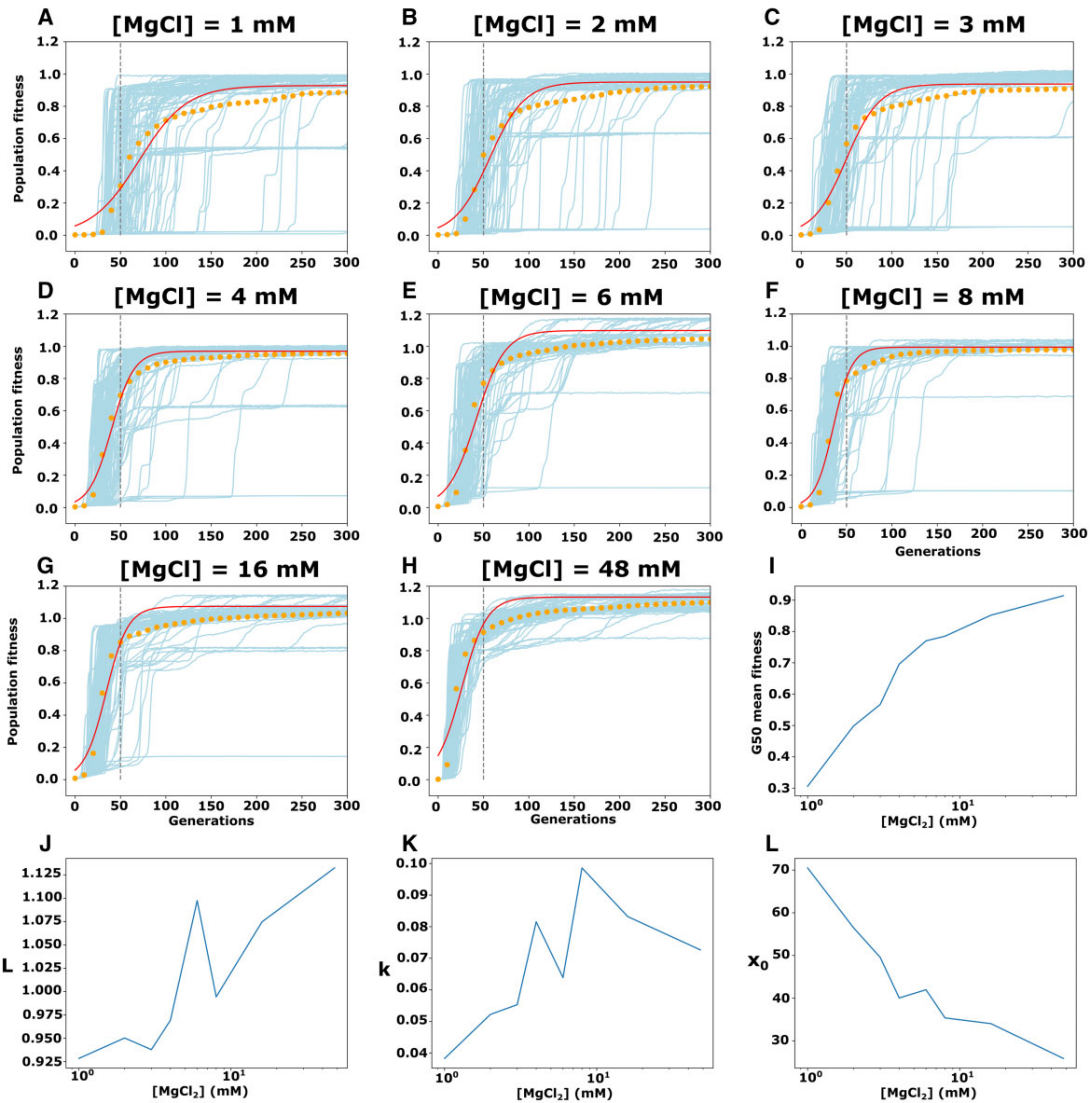
Computational simulations of populations evolving on the fitness landscapes. Evolutionary simulations were carried out with constant population size (*N* = 1,000) and mutational probability (*µ*=0.01). About 2,000 generations-long simulations were repeated 100 times on each landscape. (*A–H*) Population mean fitness (orange dots) of the 100 replicate simulations (light blue lines) on each landscape was recorded at each generation, and mean values were fit to a logistic equation: *f*(*x*)=*L*/(1 + e^(−*k*(*x*−*x*_0_))) (red lines). The best fit curves are shown for each magnesium concentration. (*I*) The average fitness of the simulated populations at generation 50 (vertical dashed lines in *A–H*). This “G50 mean fitness” value is plotted for each magnesium concentration. (*J*) Change in the maximum fitness from the logistic fitted curves compared with the change in magnesium. (*K*) Increase in the logistic growth rate (*k*) of the best-fit sigmoidal curve at each magnesium concentration. (*L*) Change in the midpoint (*x*_0_) of the best-fit curves at each magnesium concentration. Midpoints indicate the generations required to reach half the maximum fitness. Lower midpoints indicate faster adaptation.

To characterize how magnesium-induced changes in ruggedness can alter evolutionary dynamics, we measured the rates of adaptation during evolutionary simulations on the different landscapes. The simulations started with 1,000 individuals all with the same genotype. This starting genotype was chosen because it had low fitness and was seven mutations away from all the highest fitness genotypes from each magnesium concentration. The evolution of this population was simulated for 2,000 generations with a mutation rate of 0.01 and the population size was kept constant at 1,000 individuals. The average fitness of the population was recorded at each generation. For each landscape, the 2,000-generation simulation was repeated 100 times. When the 100 simulations from a specific magnesium concentration are combined, we observed a sigmoidal increase in fitness over time. As a simplified method to extrapolate the growth rate of each fitted curve, we arbitrarily set a threshold at generation 50 and recorded the mean value of population fitness at that generation for each magnesium concentration. This simplified growth rate tended to increase as the concentration of magnesium is increased (fig. 5*I*), indicating that populations were evolving faster at higher magnesium concentration. Furthermore, we fit the results from the 100 replicates at each magnesium concentration to the logistic growth equation: *f*(*x*)=*L*/(1 + e^(−*k*(*x*−*x*_0_))) (fig. 5*A–H*), where *L* is the maximum value reached by the curve, *k* is the “growth rate,” and *x*_0_ is the midpoint of the sigmoid. We found that the *L* values obtained for each landscape indicated that the populations were reaching the highest fitness genotype of the landscape by the end of the simulations (fig. 5*J*). However, the *k* values for each landscape increased with magnesium indicating a more rapid increase in fitness with higher magnesium (fig. 5*K*). The midpoints (*x*_0_) also indicated more rapid adaptation at higher magnesium. The strong decrease in *x*_0_ with increased magnesium indicates that less time (generations) was required to reach half the maximum fitness with higher magnesium. We found that the populations needed more than twice as long to reach half maximal fitness at 1 mM Mg^++^ (*x*_0_>70) as compared with 48 mM Mg^++^ (*x*_0_<30) (fig. 5*L*). These simulation results support the conclusion that the general smoothing of the landscape caused by higher magnesium can increase the rate at which populations evolve toward higher fitness.

These simulations were also repeated using randomized starting genotypes and population sized, but no notable differences were observed from the simulations with a fixed starting genotype and population size, except for growth rates being independent from the magnesium concentration, likely caused by a considerable amount of simulations starting from high-fitness genotypes (supplementary fig. 12, Supplementary Material online).

## Discussion

We have presented analysis of how a single environmental variable can change the topography of an RNA fitness landscape and alter evolutionary processes. Because the environmental variable studied impacts the stability of the active structure of these molecules, we anticipate that the general observations may be similar to other variables that alter the folding and stability of macromolecules, such as temperature, ionic strength, crowding, the presence of denaturants or molecular chaperones (Rutherford and Lindquist 1998; Desai et al. 2014; Gracia and Russell 2014; Paudel and Rueda 2014). In addition, because decreasing magnesium typically lowers RNA fitness, the data presented here can be compared with parameters in other experimental systems that increase selection pressure, such as increased antibiotics in the evolution of beta-lactamase genes (Bershtein et al. 2006).

The observation that increasing magnesium reduced ruggedness while also favoring sequences with more mutations has important evolutionary implications. The results suggest that selection under higher magnesium concentration would preserve sequences in this data set at higher mutational distances, and the reduced ruggedness means that some of the intermediates on the mutational trajectories to higher fitness would also be preserved. Together, periods of selection at higher magnesium would allow a population to explore more sequence space in this local fitness landscape. Such sequence space exploration is thought to be important for future adaptation and evolutionary innovations (Amitai et al. 2007; Wagner 2008). Although under a constant selection pressure, it is likely that populations would be attracted to local fitness peaks that disfavor sequence space exploration. Our results illustrate how changes to a single environmental variable, such as Mg^2+^ ions, can alter the future adaptive potential of an evolving population.

The results presented here demonstrate a method for high-throughput biophysical characterization of RNA. Our results show that magnesium titrations of ribozymes can be studied by deep sequencing, enabling advancements in the study of how RNA stability or folding changes with mutations. As such, our approach joins others in using sequencing technology to turn low-throughput biochemistry approaches into high-throughput approaches for characterizing RNA sequence to function relationships (Pitt and Ferré-D’Amaré 2010). For example, the deep sequencing of ribozyme reactions under varying substrate concentrations has been used to measure enzyme kinetics for vast sequence libraries (Pressman et al. 2019; Andreasson et al. 2020). Similar approaches have been used to investigate RNA sequence requirements for RNA-binding proteins (Guenther et al. 2013). One advantage of these approaches is that all sequence variants are studied simultaneously in the same reaction, ensuring identical conditions, which may improve the resolution of subtle differences between variants by reducing experimental noise. In addition, our approach used the enrichment of sequences during in vitro selection, suggesting that the analysis can be adapted to other types of selections, whether in vitro or cellular based (Townshend et al. 2015; Li and Zhang 2018). We anticipate that future experiments will combine approaches (i.e., kinetics and magnesium titrations) to better understand the interplay between the evolution of stability and catalysis.

It is informative to compare our results with recent findings of the effect of environmental change to the fitness landscapes of tRNA in yeast. Li and Zhang (2018) found that the fitness effects of mutations in a yeast tRNA were highly correlated between four different environments that altered temperature and RNA stability (3% DMSO). The comparison of fitness measurements in pairs of environments typically showed a Pearson’s correlation >0.90, enabling the prediction of fitness in one environment based on fitness measured in a different environment. In terms of RNA structure, altering magnesium ions should have a similar effect to altering temperature, in that unstable molecules will not fold properly at low magnesium or high temperature. However, in our data, we only observe such high correlations when comparing similar magnesium concentrations (supplementary fig. 5, Supplementary Material online). Some of the ribozyme variants studied here change from very low fitness to very high fitness over the magnesium concentrations studied, which contributes to low correlations. This further suggests that the magnesium concentrations used in our experiments may cover a larger range of thermodynamic “stress” than the temperature differences used in the yeast experiments (23 °C, 30 °C, or 37 °C), which is one important difference. In addition, the mutational libraries were quite different. The tRNA library used random mutagenesis to exhaustively cover single and double mutants from the wild-type sequence. The *Azoarcus* ribozyme library here focused on sequences with higher mutational distance from the wild-type, with seven random nucleotide positions and all combinations of mutations at these seven positions. If most mutations have a destabilizing effect, then it would be expected that higher mutational distances would also have more varied response to environmental changes that impact thermodynamic stability. Clearly, yeast cells contain numerous gene products that change with temperature and that could have the effect of buffering the effects of temperature on RNA structure and function. However, the differences in the library design and experimental conditions are an interesting possibility that can inspire future experiments.

Despite the high-throughput nature of our experiments, the data only explores a very small region of the total genotype space near the wild-type *Azoarcus* group I ribozyme. This local landscape data may be most informative for modeling and predicting the optimization of a known molecular function. In related work by others, the sequence space of smaller RNAs was comprehensively evaluated for their ability to catalyze a specific reaction. Such comprehensive landscapes may be more informative for understanding the origins of RNA functions (Pressman et al. 2019). This prior work found several local peaks that were separated by fitness valleys of several mutations that caused undetectable activity. There were numerous pathways to higher fitness near a local peak, but the pathways between peaks were not obviously accessible by evolutionary events, suggesting that evolution would rarely find the most optimal sequence (global peak). Our results suggest that environmental change could help bridge fitness valleys in RNA fitness landscapes, similar to what has been found in the evolution of antibiotic degrading protein enzymes studied in *Escherichia coli* during oscillating levels of antibiotics (Steinberg and Ostermeier 2016). Although both local and comprehensive fitness landscapes are informative, more work will be required to merge these two approaches in order to more comprehensively cover larger sequence spaces and form general or predictive models of the effect of environmental changes on the origins and evolution of molecular functions.

Our results join a growing of body of data that explore how fitness landscapes can be used to understand and predict evolution (de Visser and Krug 2014). The importance of the environment has been central to evolutionary theory since Darwin, and it is well accepted that changes in the environment are critical for the emergence and optimization of gene functions. Experimental fitness landscapes are now offering quantitative, molecular level insight into the processes of evolution. The sequence space of genomes, and most genes, is so vast that it will not be exhaustively explored experimentally, especially when this is combined with the combinatorial complexity of environmental variables. The goal remains to extrapolate general trends from experimental data, requiring more significant collaborations between theory and experiment. Coordinated advancements on both fronts are needed to more efficiently and accurately explore and predict functions in genotype space.

## Materials and Methods

### Library Synthesis

A DNA template was chemically synthesized with the sequence:

GTGCCTTGCGCCGGGAAACCACGCA AGGGATGGTGTCAAATTCGGCGAAACC TAAGCGCCCGCCCGGGCGTATGGCA ACGCNGAGCCAAGCT TCGGCGCCTGCGCCGATGNAGGT GTAGNGACTAGACGGCACCCACC TAAGGCNAACGNTATGGTGAAG GCATAGTCNAGGG AGNGGCGAAAG TCACACAAACCGG,

where N indicates a base synthesized with equal amounts of all four phosphoramidites (“machine mixed,” 4 nmol Ultramer, desalted, IDT). The template begins with the first three nucleotides of the ribozyme internal guide sequence (IGS) and is missing the first eight nucleotides of the natural intron sequence. This ssDNA template was PCR amplified with primers (table 1: Tas2.1a and T20a) that extend the template by 28 base pairs that include the promoter for T7 RNA polymerase. PCR reactions contained 200 µl 2× Kapa Hifi Master Mix (Roche), 12 µl of primer 1 (10 µM), 12 µl of primer 2 (10 µM), 16 µl of library DNA template (100 nM), and water to reach a reaction volume of 400 µl (8×50 µl reactions). The library DNA was amplified for 18 cycles of 98 °C for 20 s and 74 °C for 30 s, and a final extension at 72 °C for 2 min. Amplification was confirmed on a 2% agarose gel. PCR products were purified on silica-based columns (DCC, Zymo), eluted in RNase free water (IDT), and quantified by A260 (NanoDrop). The RNA was transcribed in triplicate (4 h, 37 °C) using T7 RNA polymerase (Thermo Scientific). Transcriptions were purified with TRI reagent (Direct-zol, Zymo), separated on denaturing PAGE (10%, 8M urea), excised by UV shadowing, and eluted in 0.3 M sodium acetate. The elution was filtered (0.2 µm), ethanol precipitated and resuspended in RNAse free water and quantified by A260 (Nanodrop).

### In Vitro Selections and NGS Library Preparation

A 30-nt RNA oligo was chemically synthesized with the sequence GGCAUAACUUCAAAUACUCCUCCCAACUCA (IDT). This substrate was designed to contain the compliment of the IGS (CAU), have no predicted secondary structure (NUPACK), and have a suitable primer binding Tm for reverse transcription. Ribozyme reactions were carried out in triplicate with 10 pmol of library RNA, buffer (EPPS pH 7.5) and various concentrations of added MgCl_2_ (1, 2, 3, 4, 6, 8, 16, and 48 mM). RNA was heat cooled prior to adding magnesium. The reactions were initiated with the addition of 50 pmoles substrate, incubated at 37 °C for 1 h, and stopped with EDTA equimolar to the magnesium concentration. Reacted RNA was heated to 65 °C for 5 min and cooled to room temperature with a primer (table 1: Tas1.6a-illumina) that was complementary to the substrate and added sequence compatible with NGS Unique dual index primers (IDT) to the first strand RT product. Reverse transcription (Protoscript II, NEB) reactions were incubated at 42 °C for 1 h, then 65 °C for 25 min. Second-strand synthesis was carried out by low cycle PCR (10 cycles, KAPA HiFi, Roche) using a mixture of four different forward primers (table 1: Tas2.1a-phased 1-4) to add sequence diversity and NGS Unique Dual Index compatible sequence to the second strand (Bendixsen et al. 2020). This second-strand synthesis PCR product was purified with silica-based columns (DCC, Zymo) and amplified with Illumina compatible NGS Unique Dual Index Primers (IDT). A unique index combination was used for each replicate. PCR products were quantified by qPCR and pooled equimolar. The pooled DNA was assessed with a Fragment Analyzer and sequenced on a HiSeq 4000 using a paired end 150 reads (GC3F, University of Oregon).

**Table 1.** PCR Primers Used in This Study.

Name	Sequence (5′–3′)
Tas2.1a	CTGCAGAATTCTAATACGACTCACTATAGTGCCTTGCGCCGGGAA
T20a	CCGGTTTGTGTGACTTTCGCC
Tas1.6a_illumina	GTCTCGTGGGCTCGGAGATGTGTATAAGAGACAGTGAGTTGGGAGGAGTATTTGAAGTT
Illumina-reverse	GTCTCGTGGGCTCGGAGATGTGTATAAGAGACAG
Tas2.1a_phased1	TCGTCGGCAGCGTCAGATGTGTATAAGAGACAG GCATGCATGCATGCATGC GTGCCTTGCGCCGGGAA
Tas2.1a_phased2	TCGTCGGCAGCGTCAGATGTGTATAAGAGACAG TGCATGCATGCATGC GTGCCTTGCGCCGGGAA
Tas2.1a_phased3	TCGTCGGCAGCGTCAGATGTGTATAAGAGACAG ATGCATGCATGC GTGCCTTGCGCCGGGAA
Tas2.1a_phased4	TCGTCGGCAGCGTCAGATGTGTATAAGAGACAG CATGCATGC GTGCCTTGCGCCGGGAA

### Calculating Fitness from Sequence Data Analysis

Paired end reads were joined with FLASh allowing for “outies.” The abundance of each sequence variant in the read data and curve fitting was carried out using custom python scripts available on GitLab (https://gitlab.com/haydenlab/azo7). Briefly, reads were counted that matched a regular expression of the wild-type ribozyme sequence with wild-cards at the variable positions. For each matching read, the nucleotide identity at each variable position was extracted to distinguish between the sequence variants. All sequences with mutations outside the expected positions were excluded from further analysis. For each replicate, the counts of each unique sequence variant were divided by the total count of all reads that matched to any variant. This resulted in proportion of reads represented by each sequence. Fitness was determined by dividing the proportion observed after selection in a specific magnesium concentration by the proportion observed before selection. This value was normalized to wild-type=1. The interactive data analysis tool was built with JavaScript using a custom library to enable fast rendering on the web.

### Calculating Landscape Ruggedness

Pairwise epistasis was determined for every set of four genotypes that comprise mutational “squares” in the landscapes. One genotype was defined as a reference, with a starting fitness *W*_0_. The fitness difference between this reference genotype was compared with the fitness of a genotype with two mutational differences (*W*_AB_) and each of the individual mutational differences (*W*_A_ and *W*_B_). A square was considered to have no epistasis if *W*_AB_+*W*_0_−*W*_A_−*W*_B_=0. For all the epistatic squares, two conditions were verified:

$$\text{Condition}\;1:\;\left|W_{A}–\;W_{0}+W_{\text{AB}}–\;W_{B}\right|=\left(\left|W_{A}–\;W_{0}\right|+|W_{\text{AB}}–\;W_{B}|\right)$$

$$\text{Condition}\;2:\;\left|W_{B}–\;W_{0}+W_{\text{AB}}–\;W_{A}\right|<\left(\left|W_{B}–\;W_{0}\right|+|W_{\text{AB}}–\;W_{A}|\right).$$

The squares where condition 1 was True and condition 2 was False were classified as magnitude epistasis. The squares where condition 1 is False and condition 2 is True were classified as reciprocal sign epistasis. All the remaining squares were classified as sign epistasis. The ruggedness of a landscape at a given magnesium concentration was calculated as ruggedness=2(*f*_RS_)+*f*_S_, where *f*_RS_ is the fraction of squares with reciprocal sign epistasis and *f*_S_ is the fraction of squares with sign epistasis. Roughness to slope ratio (r/s) was calculated as described previously (Aita et al. 2001). Briefly, the ratio quantifies how well each genotype fitness can be predicted from the effects of mutations on the background of the most fit genotype, assuming an additive model of fitness effects. 

## Supplementary Material

Supplementary data are available at *Molecular Biology and Evolution* online. Raw sequence data in fastq format can be found at the European Nucleotide Archive (accession number PRJEB43384).

## Supplementary Material

msab373_Supplementary_Data
