# Supplementary material for: Dynamic RNA Fitness Landscapes of a Group I Ribozyme during Changes to the Experimental Environment
Source: Mol Biol Evol. 2022 Jan 10;39(3):msab373. doi: 10.1093/molbev/msab373 (PMC8890501; doi:10.1093/molbev/msab373)
Supplement: msab373_Supplementary_Data [file msab373_supplementary_data.pdf]

Supplementary Material:

Dynamic RNA fitness landscapes of a group I ribozyme during changes to the experimental environment

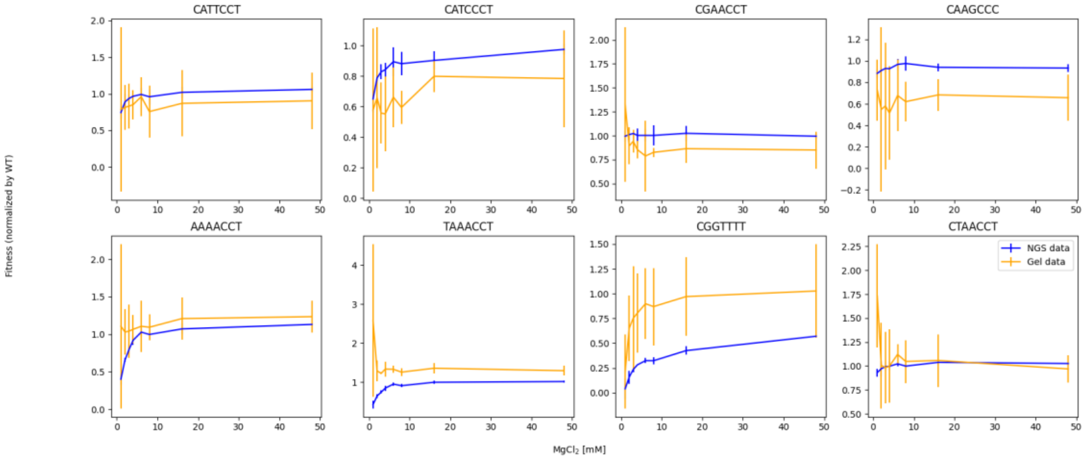

Supplementary Figure 1. Confirmation of fitness measurements of selected genotypes by gel electrophoresis. Eight genotypes of our library were individually transcribed and reacted with the RNA substrate following the same methods used for the entire library. Their activity was then evaluated by denaturing polyacrylamide gel electrophoresis. Fitness values were measured as a ratio between the amount of reacted ribozymes (intensity of the reacted band: ~230 nucleotides) and the amount of total ribozymes (sum of the intensities of the reacted and unreacted bands: ~230 and ~200 nucleotides respectively). The fitness measurements obtained by gel electrophoresis (orange lines) were normalized to wildtype and they were compared to the corresponding values measured by high-throughput sequencing (blue lines).

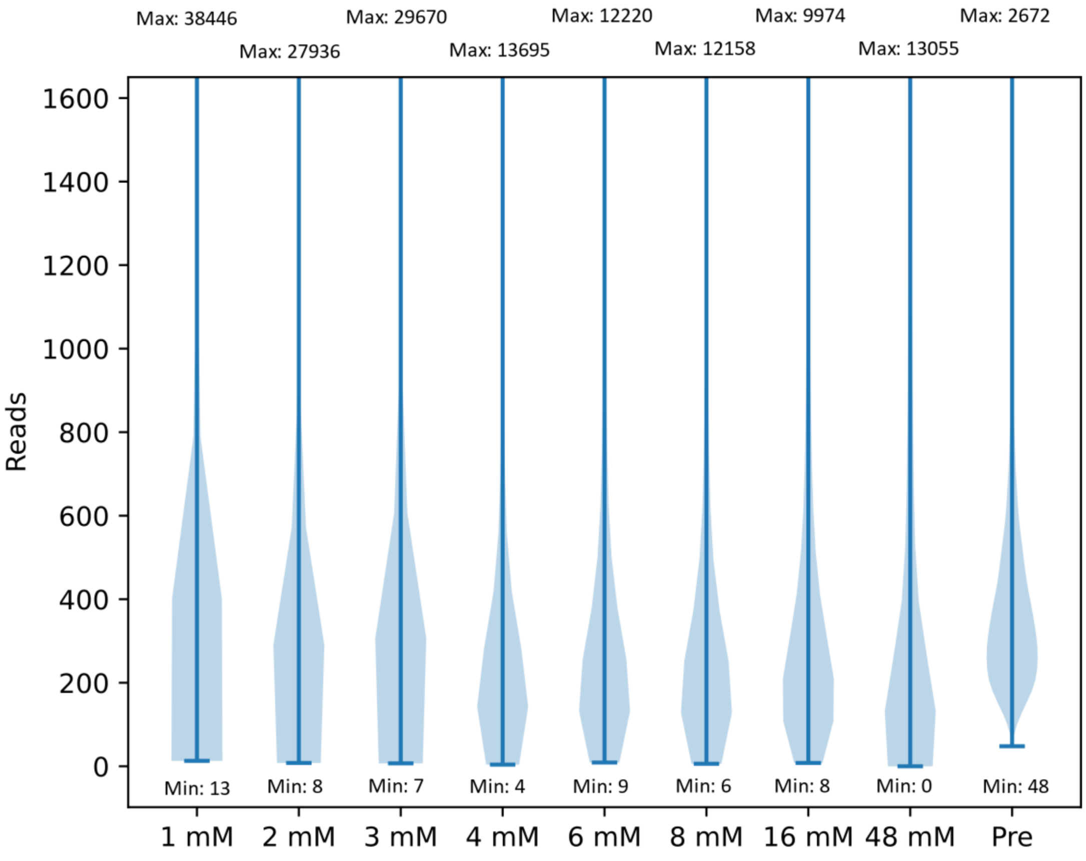

Supplementary Figure 2. Reads counted before and after activity selection. Each violin plot represents the distribution of the number of reads for each genotype counted after reaction at different magnesium concentrations (1, 2, 3, 4, 6, 8, 16, and 48 mM) and the reads for each genotype of the RNA library before activity selection (Pre). Outliers were removed for better clarity, but the maximum and minimum number of reads are shown.

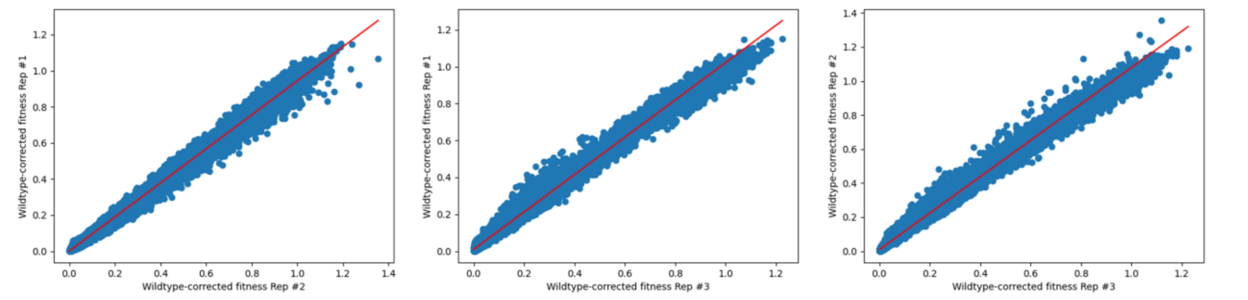

Supplementary Figure 3. Fitness correlation between replicates. These three panels represent the correlation of wildtype-corrected fitness values for each replicate pair. Left: replicates 1 and 2. Middle: replicates 1 and 3. Right: replicates 2 and 3. Each dot represents a single genotype, with its x and y coordinates corresponding to its fitness values measured in the two replicates compared. The red lines represent the linear fit of all the points of the scatter plot.

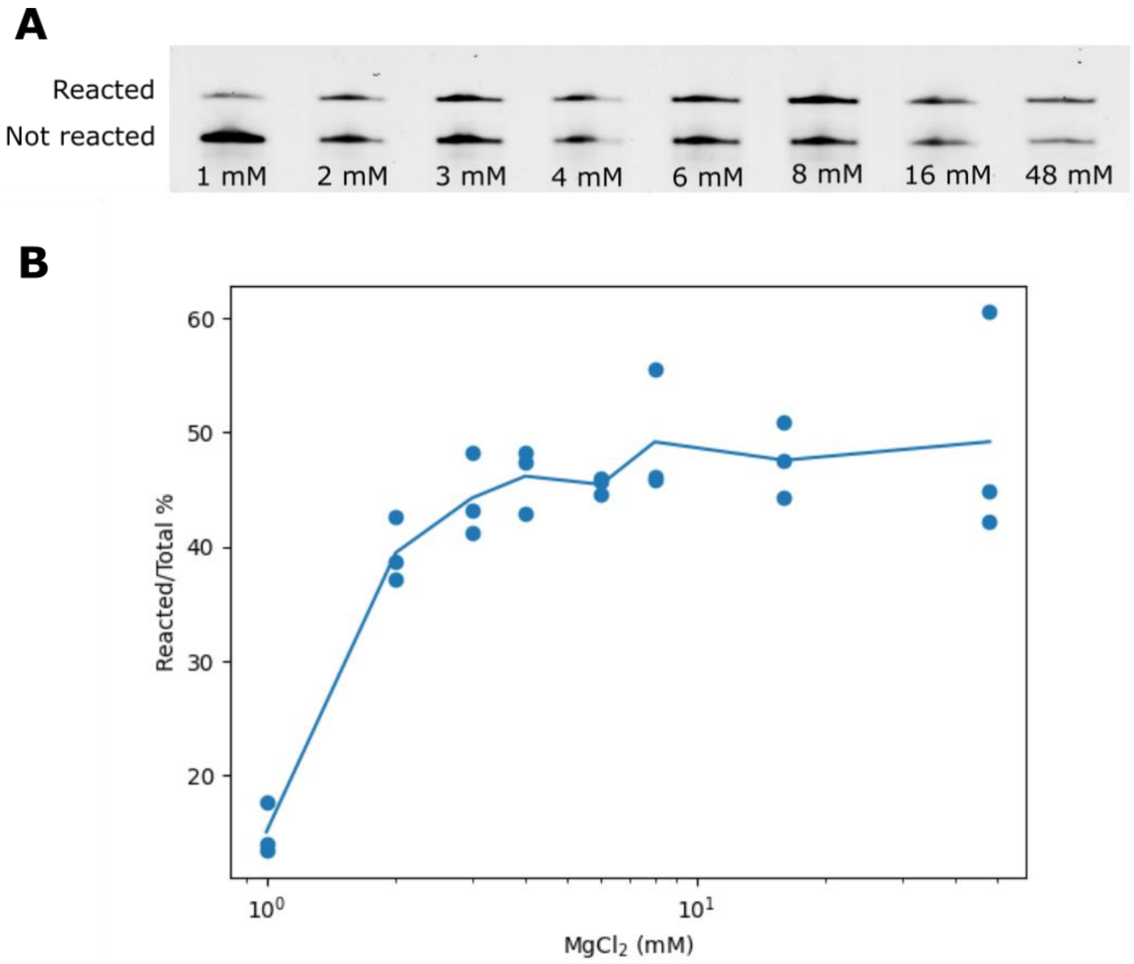

Supplementary Figure 4. Relative activity of the wild-type Azoarcus ribozyme at each magnesium concentration. A) Example polyacrylamide gel of the wild-type Azoarcus ribozyme reaction under conditions used in the sequencing data. The gel is visualized with a nucleic acid stain (GelRed, Biotium), and the inverted image is shown. B) Quantitation of **%** *Reacted* for three replicate experiments at each magnesium concentration measured by band intensities on polyacrylamide gels. The line follows the mean of the three replicates at each magnesium concentration.

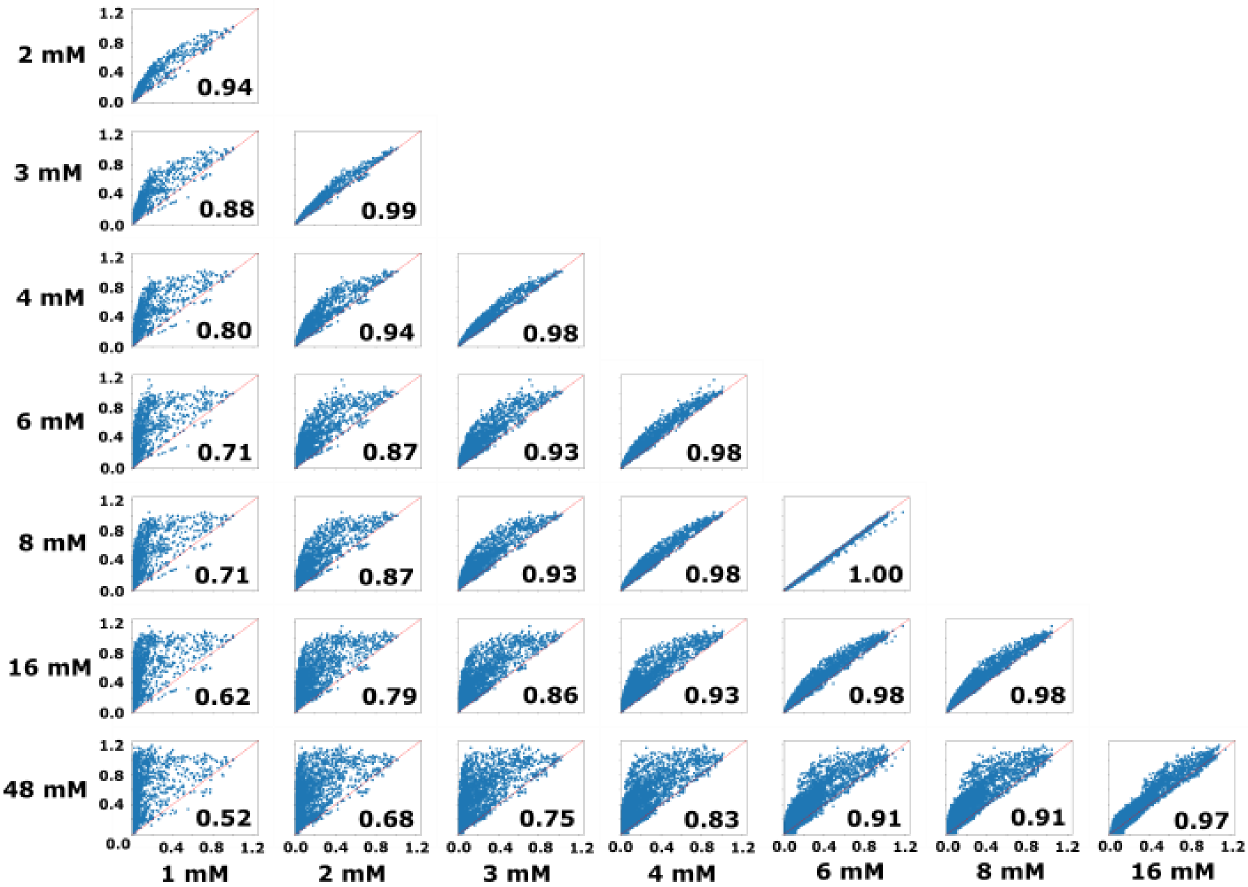

Supplementary Figure 5. Environmental effect on genotype fitness values. Comparison of the fitness of individual genotypes between all the environmental pairs. Each subplot represents the comparison between an environmental pair. Each dot represents an individual genotype with its x and y coordinates indicating its two fitness values in the environmental pair. The red line represents the diagonal.

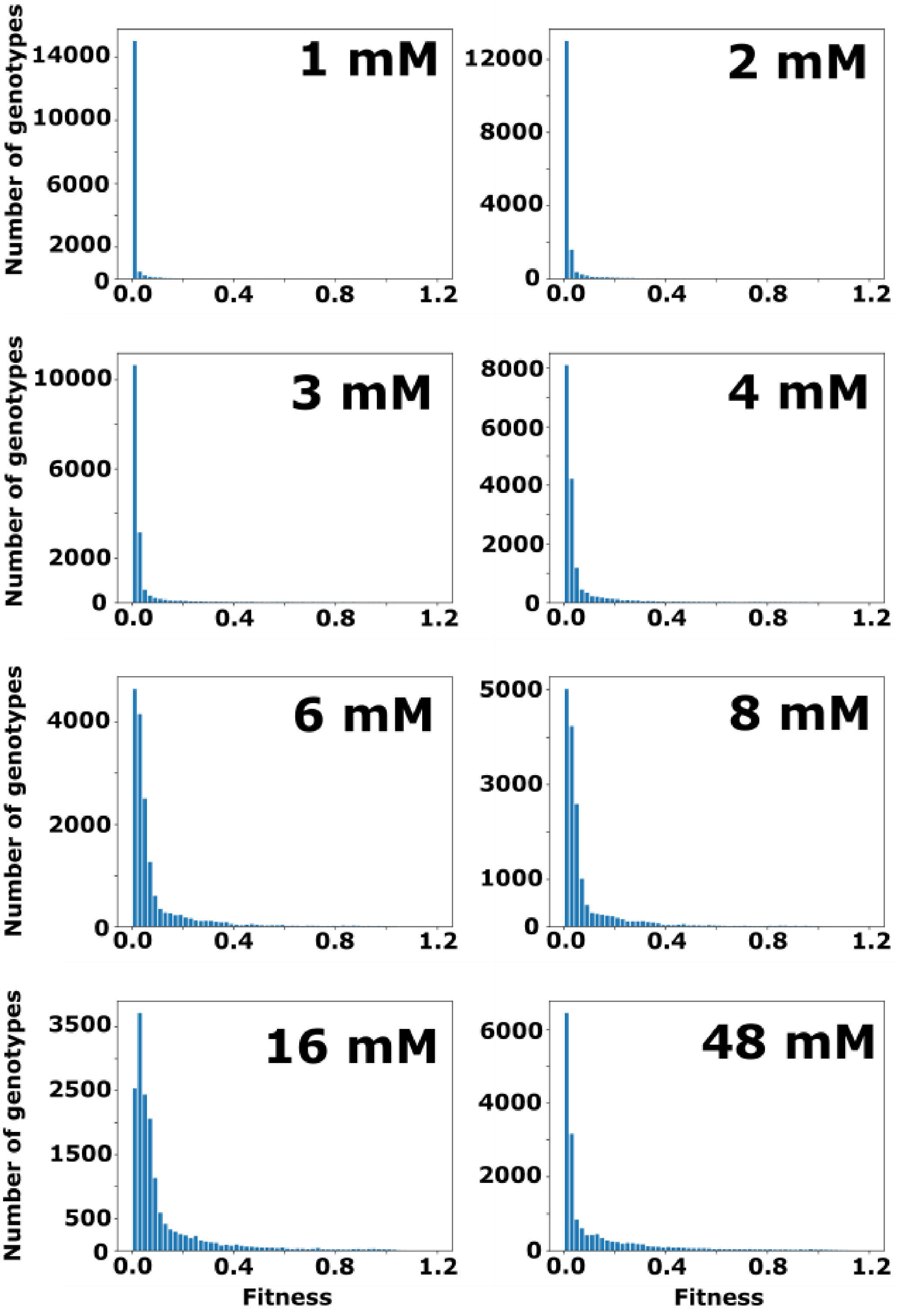

Supplementary Figure 6. Fitness distribution at different magnesium concentrations. Each subplot represents a different magnesium concentration. The height of each bar represents the number of genotypes within a certain fitness range (x axis) in a specific environmental condition (magnesium concentration).

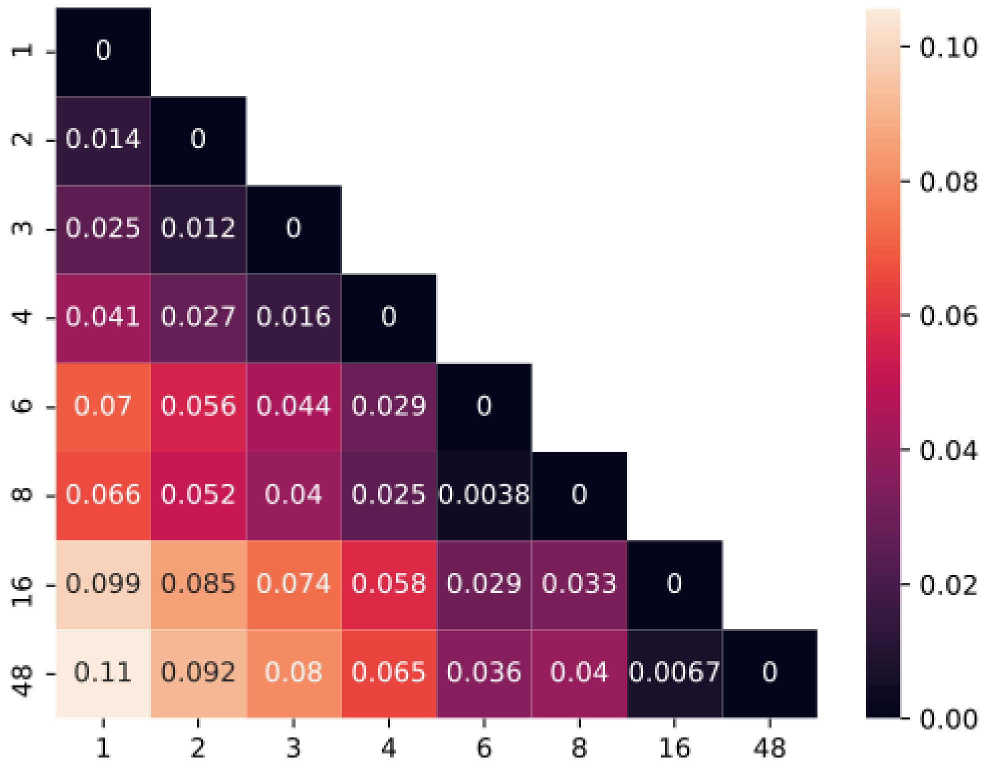

Supplementary Figure 7. Mean fitness differences between environments. Each square represents the comparison between two environmental conditions (magnesium concentration). The color intensity represents the difference between the mean fitness of the two landscapes, which is also reported numerically within the corresponding square.

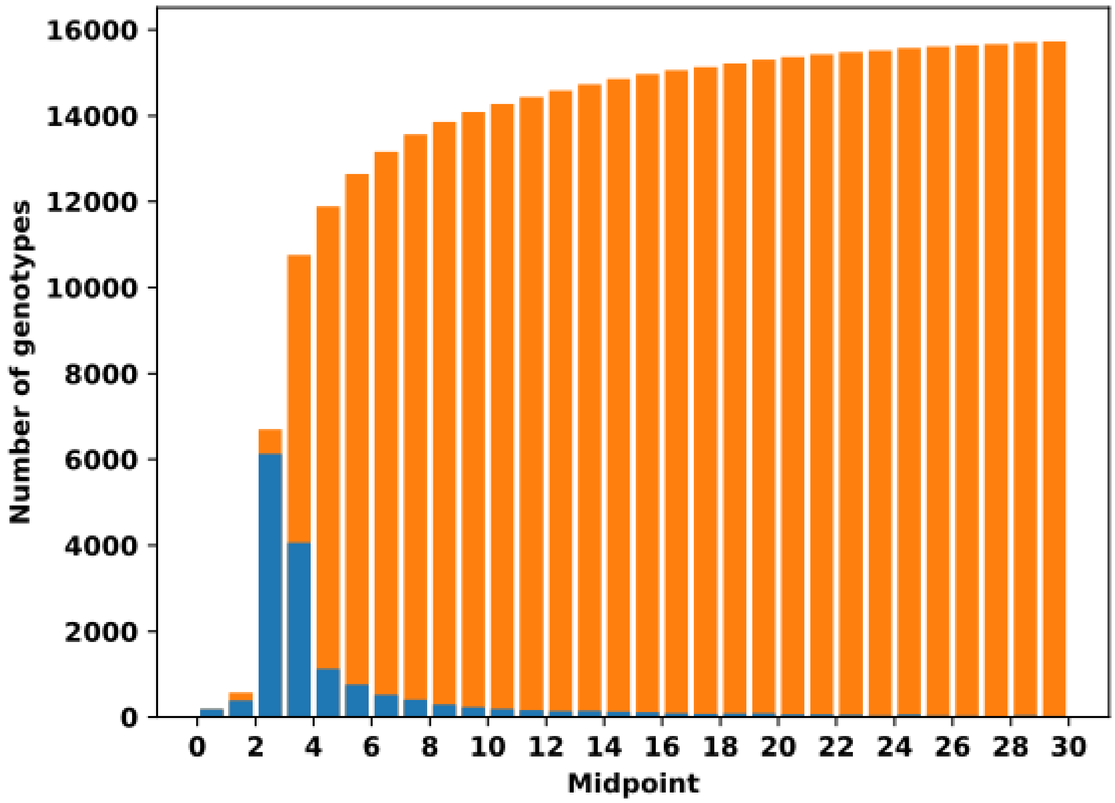

Supplementary Figure 8. Midpoint distribution. The heights of each bar represent the number of genotypes having a certain midpoint (obtained by fitting fitness values in a magnesium gradient to the Hill equation). The blue bars are the counts for each midpoint, while the orange bars are the cumulative counts.

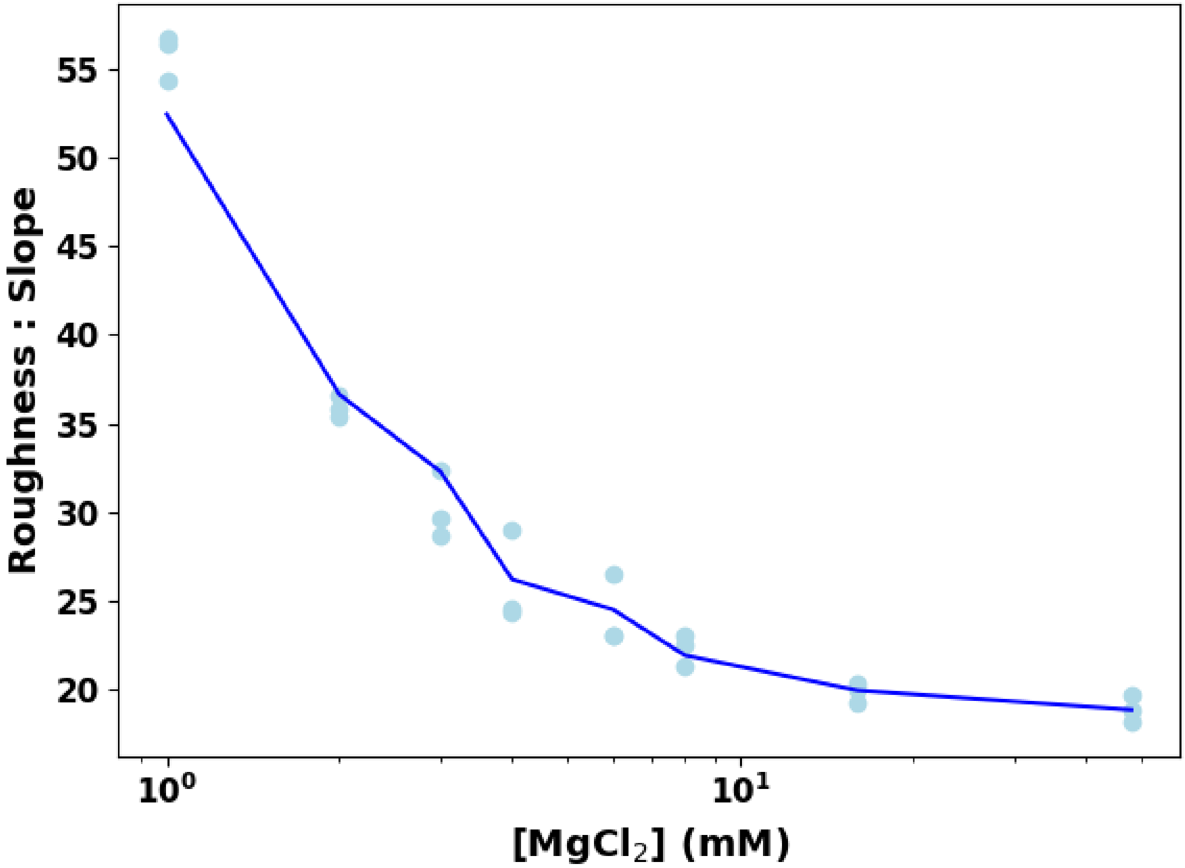

Supplementary Figure 9. Ruggedness calculated as roughness over slope (r/s). Ruggedness calculated as the ratio between "roughness" and "slope" (r/s) (Aita T., Iwakura M., Husimi Y. 2001. A cross-section of fitness landscape of dihydrofolate reductase. Protein Engineering 14: 633-638). Roughness was calculated as $\sqrt{(1/N \times \Sigma(f_{Pi} - F_{Pi})^2)}$ where N is the number of genotypes in the landscape, and f_{Pi} and F_{Pi} are the empirical fitness and its corresponding additive term respectively of ith genotype (P) of the landscape. The additive term of fitness F_{Pi} is calculated as $F_O + \Sigma w_j(\alpha_{Pj})$, where F_O is the empirical fitness of the optimal genotype ("global peak") and $w_j(\alpha_{Pj})$ is the difference in fitness caused by substituting the nucleotide at the jth position of genotype P with nucleotide α. Slope was calculated as the mean of the site fitness means ε_j, where the ε of the jth position equals $1/(\lambda_j - 1) \times \Sigma(w_j(\alpha))$, λ_j being the number of possible nucleotides at position j ($\lambda_j = 4$).

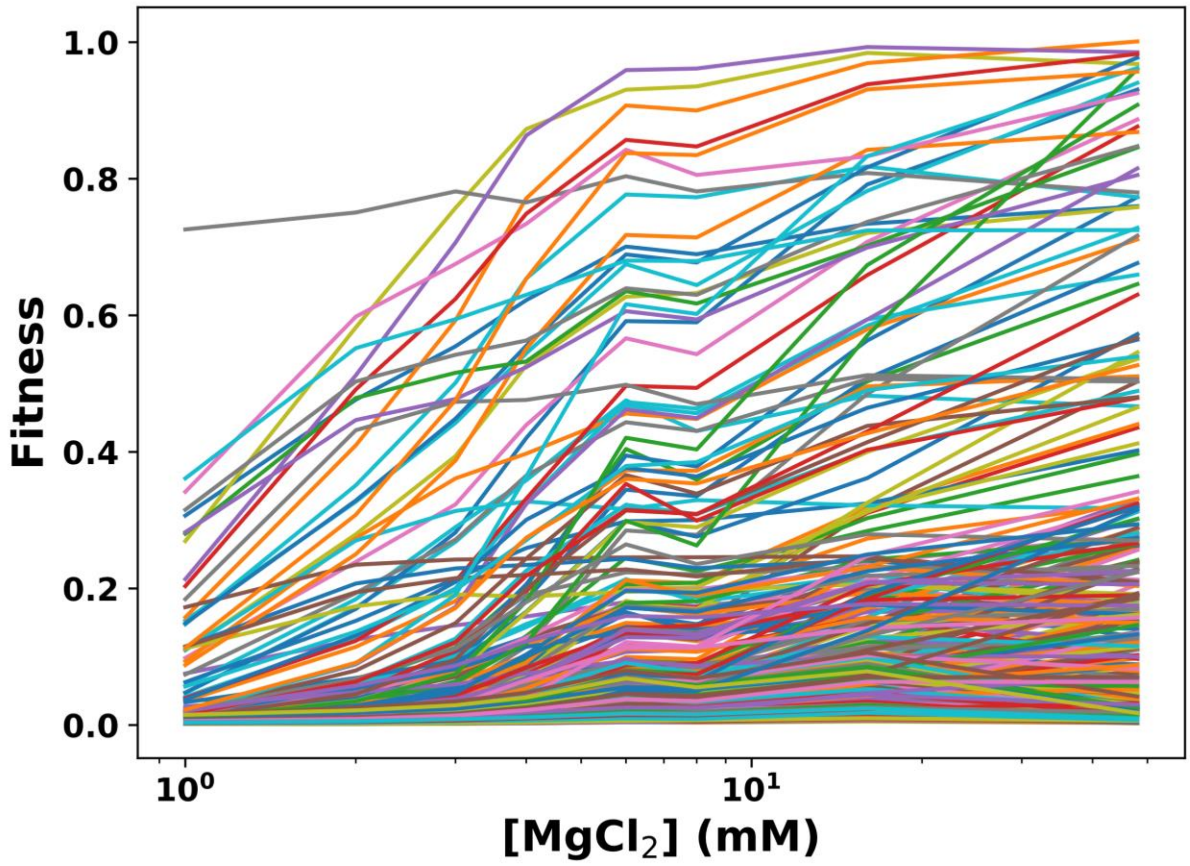

Supplementary Figure 10. Fitness fluctuations in a magnesium gradient for a selection of genotypes. 500 genotypes were randomly selected from our library of 16,384 genotypes. The x axis is the concentration of magnesium, and the y axis is the fitness of these genotypes, with each line representing the fitness trend of each individual genotype.

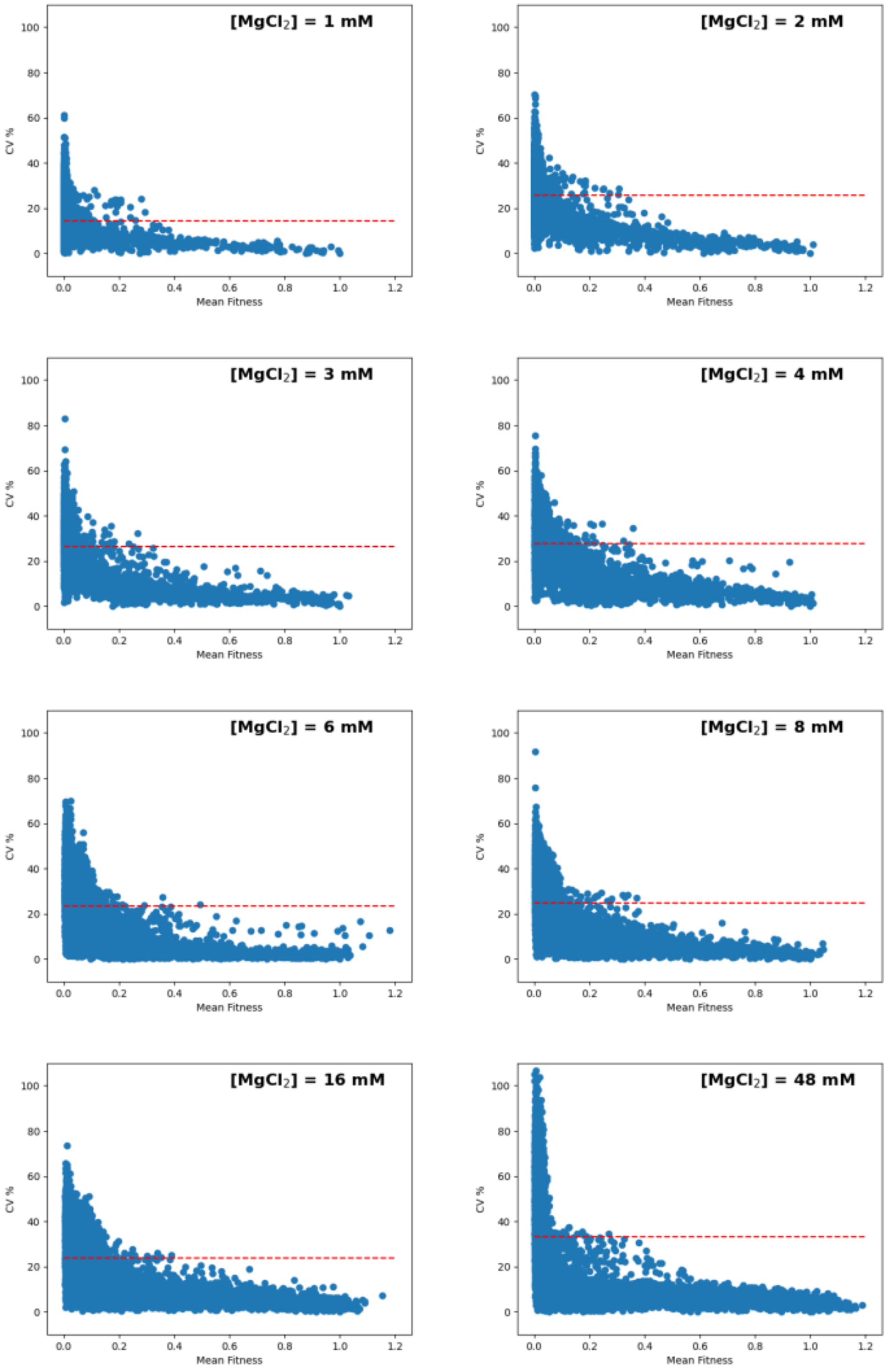

Supplementary Figure 11. Coefficient of variation between replicates. The coefficient of variation (CV) was calculated for each genotype to account for measurement error. Each panel represents a different landscape (magnesium concentration). Each blue dot represents the CV of individual genotype in relation to their measured mean fitness. The mean CV of each landscape is represented as a red dashed line.

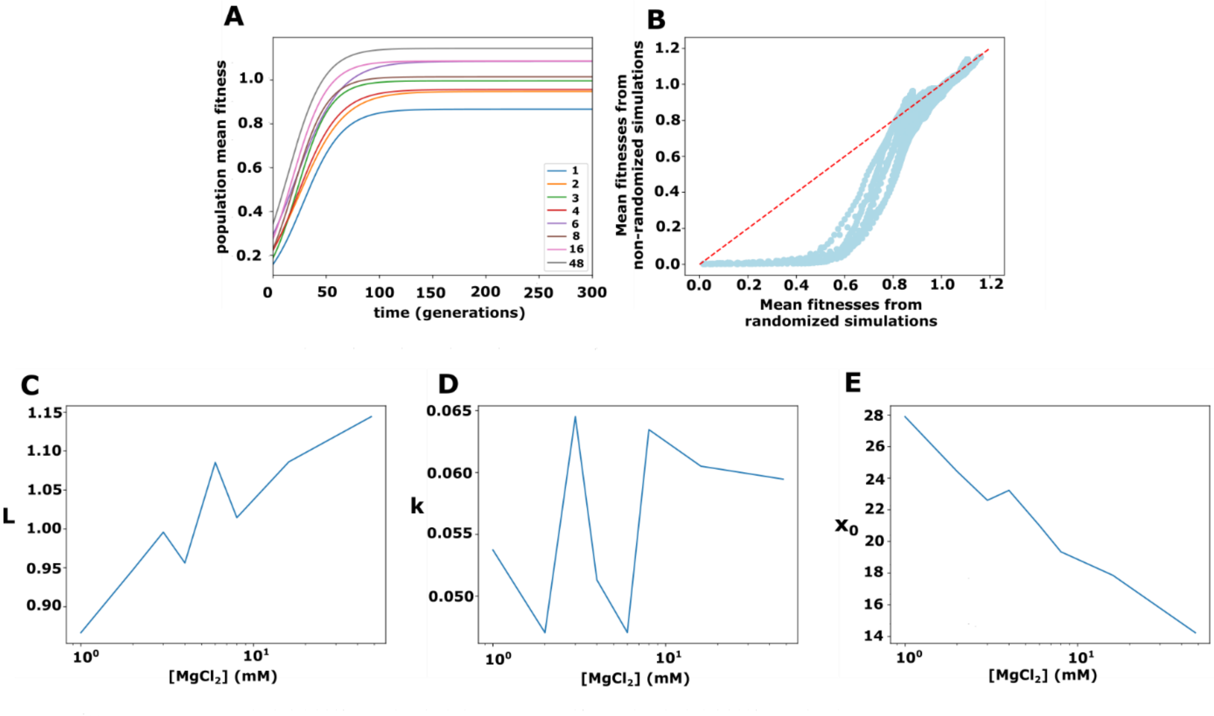

Supplementary Figure 12. Simulation effects of starting genotype and population size. The simulations of evolution were repeated using a random starting genotype (any possible genotype in the landscape) and with randomized population sizes (random number from 1 to 2000). A) Curve fitting of the mean population fitness of 100 simulations per landscape. B) Correlation plot between fixed and randomized starting genotypes and population sizes. C) Plot representing the trend of the max fitness (L) in a magnesium gradient. D) Plot representing the trend of the slope (k) in a magnesium gradient. (E) Plot representing the trend of the midpoint (x_0) in a magnesium gradient.
